# Lessons for Medicare Part D in the hemodialysis community

**DOI:** 10.1186/1471-2369-7-11

**Published:** 2006-07-06

**Authors:** Anisa I Nayeem, Glenn M Chertow

**Affiliations:** 1 Division of Nephrology, Departments of Medicine, University of California San Francisco, San Francisco, CA, USA; 2 Epidemiology and Biostatistics, University of California San Francisco, San Francisco, CA, USA

## Abstract

**Background:**

Medicare beneficiaries without prescription drug coverage consistently fill fewer prescriptions than beneficiaries with some form of drug coverage due to cost. ESRD patients, who are disproportionately poor and typically use multiple oral medications, would likely benefit substantially from any form of prescription drug coverage. Because most hemodialysis patients are Medicare-eligible, they as well as their providers would be expected to be well informed of changes in Medicare prescription drug coverage. By examining the level of understanding and use of the temporary Medicare Prescription Drug Discount Card Program in the hemodialysis population, we can gain a better understanding of the potential long-term utilization for Medicare Part D.

**Methods:**

We surveyed English-speaking adult hemodialysis patients with Medicare coverage from two urban hemodialysis centers affiliated with the University of California San Francisco (UCSF) during July and August 2005 (n = 70). We also surveyed University- and community-based nephrologists and non-physician dialysis health care professionals over the same time frame (n = 70).

**Results:**

Fifty-nine percent of patients received prescription drug coverage through Medi-Cal, 20% through another insurance program, and 21% had no prescription drug coverage. Forty percent of patients with no prescription drug coverage reported "sometimes" or "rarely" being able to obtain medications vs. 22% of patients with some form of drug coverage. None of the patients surveyed actually had a Medicare-approved prescription drug card, and of those who intended to apply, only 10% reported knowing how to do so. Only 11% health care professionals knew the eligibility requirements of the drug discount cards.

**Conclusion:**

Despite a significant need, hemodialysis patients and providers were poorly educated about the Medicare Prescription Drug Discount Cards. This has broad implications for the dissemination of information about Medicare Part D.

## Background

Medicare beneficiaries without prescription drug coverage have considerable difficulty filling prescriptions due to costs. The Kaiser Family Foundation found that 26% of seniors reported medication non-adherence due to cost, including 35% of those with three or more chronic conditions [1]. Medicare beneficiaries without drug coverage filled one-third fewer prescriptions in 2002 than beneficiaries with some form of drug coverage – a difference that persisted across a range of individual characteristics, including health status and income [1].

On December 8, 2003, President George W. Bush signed the Medicare Prescription Drug Improvement and Modernization Act of 2003 (the "MMA") into law, which added a prescription drug benefit to the Medicare program. The Medicare Prescription Drug Discount Card and Transitional Assistance Program were intended as temporary measures to provide immediate assistance in lowering prescription drug costs for persons in need. The law made Medicare-approved Prescription Drug Discount Cards available to Medicare beneficiaries as of May 2004, in place until Medicare Part D prescription drug coverage began on January 1, 2006 [2].

The net federal cost of Medicare Part D is estimated to be $37 billion in 2006, increasing to $67 billion in 2010, and totaling $724 billion for the decade of 2006–2015 [1]. Early reports suggested that the education of the public lagged prior to implementation of Medicare Part D. A survey conducted by the Kaiser Family Foundation August 4^th ^through 8^th^, 2005, including 300 adults age 65 years or older, found that only 33% of seniors felt that they had enough information at that time about the new Medicare prescription drug benefit to understand how it would affect them personally, and only 22% of seniors responded that they would enroll in a Medicare drug plan when available. Of those who said that they would not enroll or were not yet informed enough to make a decision, 26% felt that the system was too complicated. Only about 7% had called the Medicare hotline and 6% stated that a friend or family member had called the Medicare hotline or visited the Medicare website on their behalf [3]. A USA Today/CNN/Gallup poll of 275 adults age 65 years or older taken at the end of September 2005 reported that 37% of seniors stated that they understood the program at least somewhat well, while 61% did not. Only 24% of seniors surveyed stated that they planned to join Medicare Part D, compared with 54% who did not plan to sign on [4].

Persons with end-stage renal disease (ESRD) are among those for whom *per capita *Medicare spending is highest – $64,614 on average in 2003 [5] – and are disproportionately poor. Moreover, ESRD patients typically use multiple oral medications, including drugs for diabetes mellitus, heart failure, hypertension, hyperphosphatemia and other associated conditions. Patients with ESRD are Medicare-eligible before the age of 65 years, and as a function of hemodialysis, are regularly in contact with physicians, nurses, dietitians and social workers. Thus, hemodialysis patients in particular should be informed of this program and utilize it where appropriate.

Much can be learned about the potential long-term utilization of Medicare Part D in the hemodialysis population from examining the level of understanding and use of the temporary Medicare Prescription Drug Discount Cards. More than 7 million Medicare beneficiaries were eligible for these cards, which were initially projected to offer discounts of 10–25% [6]. A drug report from the Centers for Medicare and Medicaid Services (CMS) released October 12, 2004, reported that beneficiaries could obtain discounted prices 12–21% less than national average prices paid by all Americans for commonly used brand name drugs at retail pharmacies, with even larger discounts (28–75%) on generic drugs [7]. Despite multiple projections of potential cost savings, no studies to date have determined the actual benefit of the temporary Medicare Prescription Drug Discount Cards accrued to individuals. Information from such an inquiry would be valuable to the ESRD patient and provider communities, as well as more generally to the Medicare population.

To explore knowledge and utilization of Medicare Prescription Drug Discount Cards, we surveyed a sample of hemodialysis patients from two urban University-affiliated dialysis centers and a sample of dialysis care providers. We hypothesized that the levels of understanding and use of these cards would be relatively low, despite a significant need.

## Methods

We surveyed hemodialysis patients from two urban hemodialysis centers affiliated with the University of California San Francisco (UCSF) during July and August 2005. Non-English-speaking individuals were not approached. San Francisco General Hospital Dialysis Unit largely serves a disenfranchised patient population with a high proportion of minorities. The UCSF-Mt. Zion Dialysis Unit serves an ethnically diverse, insured patient population. Patients were approached in person and asked to complete the surveys while on hemodialysis. We asked for information on current medical insurance coverage, prescription drug coverage, ability to pay for medications and the use of Medicare-approved Prescription Drug Discount Cards. A total of 74 of 80 (93%) patients approached returned completed surveys; four surveys were incomplete and were not included. The most common reason for nonparticipation was "feeling too tired" to complete the survey during hemodialysis.

We also surveyed a group of University- and community-based nephrologists preceding a UCSF Nephrology Grand Rounds, and non-physician dialysis health care professionals at dialysis centers over the same time frame. A total of 72 of 74 (97%) health care professionals returned surveys, two of which were incomplete and not included in the analysis. The most common reason for nonparticipation was "too busy."

Due to the small sample size and primarily descriptive nature of the study, formal statistical testing was not pursued. The study was approved by the UCSF Committee on Human Research.

## Results

Patient survey results are presented in Table 1. The majority of participants were dually covered by Medicare and Medi-Cal (California's Medicaid). Fifty-nine percent of patients received prescription drug coverage through Medi-Cal, 20% received benefits through another insurance program, and 21% had no prescription drug coverage at all. Forty (57%) patients reported that they could always obtain all of their medications. Of the 40, 20 (50%) received prescription drug coverage through Medi-Cal and 12 (30%) through other insurance coverage. Forty percent (6/15) of patients with no prescription drug coverage reported that they were obtain their medications "sometimes" or "rarely" vs. 22% (16/55) of patients with some form of drug coverage. None of the patients surveyed actually had a Medicare-approved prescription drug card, and of those who intended to apply, only 3 (10%) reported knowing how to do so.

**Table 1:** Patient survey results

	N = 70 (%)
SFGH	37 (53%)
Mt Zion	33 (47%)
"Do you have health insurance in addition to Medicare?"	
Medical	43 (61%)
Insurance through employer	6 (9%)
Insurance through private plan	10 (14%)
No other insurance	11 (16%)
"Do you have prescription drug coverage?"	
Medi-Cal	41 (59%)
Medicare-approved prescription discount card	0 (0%)
Through another drug benefit program	14 (20%)
No prescription drug coverage	15 (21%)
"Please mark the statement that best describes your current ability to obtain your prescription medications:"	
I can always obtain all of my meds (100% of the time)	40 (57%)
I can usually obtain all of my meds (60–99%)	18 (26%)
I can sometimes obtain all of my meds (30–59%)	11 (16%)
I can rarely obtain all of my meds (<30%)	1 (1%)
"In the past 12 months, have you been unable to take a medication that was prescribed to you because you could not afford it?"	
Yes	21 (30%)
No	49 (70%)
"Do you have a Medicare Prescription Drug Card?"	
Yes	0 (0%)
No	70 (100%)
"Do you intend to apply for a Medicare Prescription Drug Card?"	
Yes	31 (44%)
No	39 (56%)
"Do you know how to apply for a Medicare Prescription Drug Card?"	
Yes	8 (11%)
No	62 (89%)
"Would you be interested in learning more about the Drug Cards?"	
Yes	57 (81%)
No	13 (19%)

The health professional survey results are presented in Table 2. Only eight (11%) health care professionals knew the eligibility requirements of the drug discount cards, including only one of 17 (6%) nephrologists who care for hemodialysis patients >20% of their clinical time. Only five (7%) providers knew the eligibility requirements of the low income subsidy, including none of the nephrologists. A small fraction of nephrologists were ever asked about the Medicare prescription drug discount card by patients, and even fewer could provide their patients with relevant information (Table 2). Finally, of the ten providers that knew how to obtain a card, nine (90%) counseled at least one patient to apply.

**Table 2:** Health care professionals survey results

	N = 70 (%*)
Field of professionals-	
Nephrologists with > 20% of time caring for HD patients**	16 (23%)
Nephrologists with < 20% of time caring for HD patients	11 (16%)
Nurse practitioner with > 20% of time caring for HD patients**	1 (1%)
Social worker	3 (4%)
Nurse/dialysis technician	32 (46%)
Dietician	1 (1%)
Pharmacist	3 (4%)
Other	3 (4%)
"Do you know the eligibility requirements of the Medicare Prescription Drug Discount Cards?"	
Yes	8 (11%)
No	62 (89%)
"Do you know the eligibility requirements of the low income subsidy?"	
Yes	5 (7%)
No	65 (93%)
"Do you know which cards are available in your state/region?"	
Yes	9 (13%)
No	61 (87%)
"Do you know how to get a card?"	
Yes	10 (14%)
No	60 (86%)
"Have you ever counseled a patient to apply for a card?"	
Yes	4 (6%)
No	66 (94%)
"Have you ever been asked by a patient which card you would recommend?"	
Yes	4 (6%)
No	66 (94%)
"Have you ever been asked by a patient how to get a card?"	
Yes	8 (11%)
No	62 (89%)
"Would you be interested in learning more about the Medicare Prescription Drug Discount Card?"	
Yes	54 (77%)
No	16 (23%)

*May not equal 100 due to rounding error

**Combined in further analyses due to small number of nurse practitioners

## Discussion

Medicare Part D is an ambitious attempt to help Medicare beneficiaries reduce out-of-pocket expenses for prescription drugs. The average per capita drug spending among Medicare beneficiaries in 2005 is estimated to be $2,864 [1]. Hemodialysis patients, by the nature of their disease, often have extensive needs for prescription drugs. The results described here support the observation that lack of prescription drug coverage leads to difficulty in obtaining necessary medications. The novel findings of this study are 1) persons on hemodialysis had scarce knowledge about the Medicare-approved Prescription Drug Discount Cards, despite the need for multiple medications and suboptimal drug coverage; 2) discount cards were underutilized in this group (zero of 70 patients); and 3) health care professionals who care for a largely Medicare-eligible population demonstrated an egregious lack of knowledge about this program.

Several caveats are worth noting. The patients surveyed were from urban dialysis centers, and may not reflect the knowledge base of the overall hemodialysis or home peritoneal dialysis populations. However, we selected English-speaking patients; we anticipate that non-English-speaking patients may experience even more difficulty navigating the system. It is possible that patients and providers in for-profit dialysis facilities may be better informed, though many of the providers surveyed in this study work jointly in independent and for-profit dialysis facilities.

The educational and outreach efforts of CMS to date have included information in 14 languages on the Medicare website and through the 1-800-MEDICARE line, a brochure for beneficiaries, a national multi-media campaign, and a section in the *Medicare & You 2005 *handbook [8]. Despite the availability of these materials, and the existence of the Medicare Prescription Drug Discount Card Program for over a year, hemodialysis patients and providers remained poorly educated about the Medicare Prescription Drug Discount Cards. This has broad implications for the dissemination of information about Medicare Part D. While actions will speak louder than words, the survey results suggest that providers would be willing to assist patients in obtaining drug discount cards once adequately informed.

The Kidney Medicare Drugs Awareness and Education Initiative was launched in Washington, D.C. on July 27, 2005 in an effort to inform patients with chronic kidney disease about Medicare Part D. This initiative includes more than 35 kidney organizations and companies, led by the National Kidney Foundation (NKF). The centerpiece of this initiative is a new website for patients and professionals [9]. The efforts of the CMS to bring information to the general public about Medicare Part D include information in the *Medicare & You 2006 *handbook, the new "search tool" option on the Medicare website, the 1-800-MEDICARE line [10], and an ad insert explaining the program in the September 25, 2005, edition of *Parade*, a Sunday newspaper supplement with a circulation of 34.5 million people. In addition, the American Association of Retired Persons (AARP), the nation's largest advocacy group for seniors, has sponsored education campaigns.

## Conclusion

Of the 43.1 million current Medicare beneficiaries, 29.3 million (70%) are expected to enroll in Medicare Prescription drug plans over the course of this year according to the Administration [1]. Members of the dialysis provider communities must be better educated, so that dialysis patients have the information and tools to take advantage of these programs.

## Competing interests

The author(s) declare that they have no competing interests.

## Authors' contributions

AIN conceived of the study, participated in the design of the study, and helped to draft the manuscript. GMC participated in the design of the study and helped to draft the manuscript. Both authors read and approved the final manuscript.

## Pre-publication history

The pre-publication history for this paper can be accessed here:


